# The Radiological Physics Center's standard dataset for small field size output factors

**DOI:** 10.1120/jacmp.v13i5.3962

**Published:** 2012-08-08

**Authors:** David S. Followill, Stephen F. Kry, Lihong Qin, Jessica Leif, Andrea Molineu, Paola Alvarez, Jose Francisco Aguirre, Geoffrey S. Ibbott

**Affiliations:** ^1^ Department of Radiation Physics Radiological Physics Center The University of Texas M. D. Anderson Cancer Center Houston Texas USA; ^2^ Department of Therapeutic Radiology University of Minnesota Minneapolis MN USA

**Keywords:** IMRT, photon dosimetry, small field size dependence, quality assurance, output factors

## Abstract

Delivery of accurate intensity‐modulated radiation therapy (IMRT) or stereotactic radiotherapy depends on a multitude of steps in the treatment delivery process. These steps range from imaging of the patient to dose calculation to machine delivery of the treatment plan. Within the treatment planning system's (TPS) dose calculation algorithm, various unique small field dosimetry parameters are essential, such as multileaf collimator modeling and field size dependence of the output. One of the largest challenges in this process is determining accurate small field size output factors. The Radiological Physics Center (RPC), as part of its mission to ensure that institutions deliver comparable and consistent radiation doses to their patients, conducts on‐site dosimetry review visits to institutions. As a part of the on‐site audit, the RPC measures the small field size output factors as might be used in IMRT treatments, and compares the resulting field size dependent output factors to values calculated by the institution's treatment planning system (TPS). The RPC has gathered multiple small field size output factor datasets for X‐ray energies ranging from 6 to 18 MV from Varian, Siemens and Elekta linear accelerators. These datasets were measured at 10 cm depth and ranged from $10\times 10\;{\text{cm}}^{2}$ to $2\times 2\;{\text{cm}}^{2}$. The field sizes were defined by the MLC and for the Varian machines the secondary jaws were maintained at a $10\times 10\;{\text{cm}}^{2}$. The RPC measurements were made with a micro‐ion chamber whose volume was small enough to gather a full ionization reading even for the $2\times 2\;{\text{cm}}^{2}$ field size. The RPC measured output factors are tabulated and are reproducible with standard deviations (SD) ranging from 0.1% to 2.4%, while the institutions' calculated values had a much larger SD range, ranging up to 7.9%. The absolute average percent differences were greater for the $2\times 2\;{\text{cm}}^{2}$ than for the other field sizes. The RPC's measured small field output factors provide institutions with a standard dataset against which to compare their TPS calculated values. Any discrepancies noted between the standard dataset and calculated values should be investigated with careful measurements and with attention to the specific beam model.

PACS number: 87.53.Bn

## I. INTRODUCTION

Modern radiotherapy routinely involves the use of small radiation fields, either for the delivery of stereotactic treatments, or as components of intensity‐modulated radiation therapy (IMRT). However, quantifying and accounting for the associated field size dependent output factors for such small fields poses several challenges.^(1)^

First, commissioning a treatment planning system for such small fields poses many unique challenges. Notably, planning system accuracy for small fields is often more sensitive to modeling than for large fields. In particular, source size^2^, ^3^, ^4^ and MLC modeling^(5)^ impact dose calculation for small fields more so than for larger fields. As IMRT and stereotactic radiotherapy typically use many small segments or fields to achieve the desired dose distribution and target coverage, accurate small field size dependent output factors are required to be modeled within the TPS.

Second, complicating the issue of commissioning small radiotherapy fields is the challenge in making accurate small field dose measurements. The challenges of penumbra size versus detector size^6^, ^7^ and the impact of changes in the energy spectrum on detector response^6^, ^7^, ^8^ all complicate the measurement process. Consequently, there have been multiple incidents recently of incorrect small field size output factors being measured resulting in the mistreatment of patients.^(9)^

Because of the difficulties in commissioning small field data, a set of field size dependent output factors could prove to be an invaluable tool to confirm the validity of an individual institution's dosimetry parameters, as well as possibly identify potential dosimetry parameter discrepancies. To the authors' knowledge, no such database of field size dependent output factors exists. However, the Radiological Physics Center is well‐situated to generate such data based on a broad range of measurements on many different linear accelerators.

The Radiological Physics Center's mission for the past 43 years has been to assure the National Cancer Institute (NCI) and its funded cooperative clinical trial study groups that participating institutions deliver comparable and consistent radiation doses to clinical trial patients. In order to accomplish its mission, the RPC performs a series of quality audits both remotely and on‐site. The RPC's on‐site dosimetry review visits (site visits) consist of a series of ionization measurements (including calibration, depth dose data, wedge factors, etc.) to compare measured dosimetry parameters to the institution's treatment planning system's (TPS) calculated dosimetry parameters that are used for patient monitor unit calculations. The RPC also measures output factors as a function of field size, including small field sizes defined by the multileaf collimator (MLC), to assess whether an institution's dose calculations are accurate for small field sizes that might be used as segments within an IMRT dose distribution or stereotactic treatment.

The current study presents measured MLC‐defined small field output factors for the three major linear accelerator manufacturers and for a variety of X‐ray energies. These data offer the radiotherapy community an independent and consistently measured set of small field output factors, as measured by the RPC, to be used as a secondary QA dataset.

## II. MATERIALS AND METHODS

### A. Measurements

Small field output factors were measured by the RPC as part of on‐site quality audits at North American institutions participating in NCI clinical trials. Measurements were made on modern Varian (n=64) (Varian Medical Systems, Palo Alto, CA), Elekta (n=22) (Elekta, Stockholm, Sweden), and Siemens (n=10) (Siemens AG, Munich, Germany) accelerators that included an MLC and had indicated clinical use of IMRT. Measurements were made on available photon energies used or commissioned for IMRT (6 MV–18 MV).

**Figure 1:**
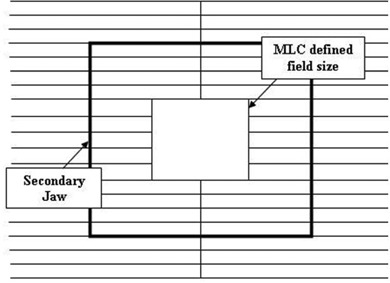
Example of the field size definition for a Varian accelerator. The secondary jaws kept fixed at a 10 X $10\;{\text{cm}}^{2}$ and the various small field sizes defined by the MLC.

The MLC shaped fields were defined at 100 cm SSD and the point of measurement was at an effective depth of 10 cm in water. The field sizes for the Siemens and Elekta linear accelerators were defined by the secondary jaws that included an MLC. The field sizes for the Varian accelerators were defined by the tertiary MLC, while the secondary jaws were kept fixed at $10\times 10{\;\text{cm}}^{2}$, as seen in Fig. 1. This configuration presented a realistic jaw/MLC configuration for IMRT, and also maximized the impact of MLC modeling within the treatment planning system by maximizing the ratio of secondary jaw opening to MLC opening. Ionization was measured for a $10\times 10{\;\text{cm}}^{2}$ field, as well as for 6×6, 4×4, 3×3 and $2\times 2\;{\text{cm}}^{2}$ fields. Field size dependence output factors were normalized to the $10\times 10{\;\text{cm}}^{2}$ field value.

**Figure 2:**
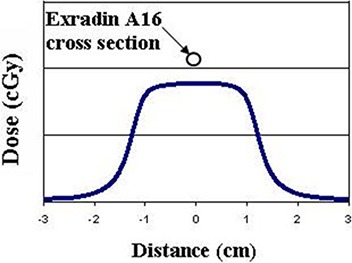
Dose profile at a depth of 10 cm for a Varian $2\times 2{\;\text{cm}}^{2}$ MLC‐shaped field size and an axial cross section of an Exradin A16 microchamber used in the RPC measurements.

Measurements were made in a rectangular, custom‐built, RPC water phantom positioned at 100 cm SSD. The smallest dimension of the water phantom was at least 25 cm. Ionization was measured using a commercially available Exradin model A16 cylindrical microchamber ($0.007\;{\text{cm}}^{3}$ sensitive volume) ion chamber (Standard Imaging, Madison, WI) connected to a Max 4000 electrometer (Standard Imaging, Madison, WI). Figure 2 shows an RPC measured dose profile at a depth of 10 cm for the smallest field size ( $2\;\times \;2\;{\text{cm}}^{2}$) in water with the axial cross‐section view of the Exradin A16 ion chamber. Figure 2 illustrates that the microchamber is sufficiently small enough to fall within the flat portion of the dose distribution, allowing it to make an appropriate ionization measurement without any loss of signal because of dose falloff near the edge of the field. Moreover, in comparisons between detectors, pinpoint ion chambers with double the active volume have been shown to be accurate within 1%, compared to other small field detectors (including diamond and diodes), down to $2\times 2{\;\text{cm}}^{2}$ fields.^6^, ^7^, ^10^ The Exradin A16 was always equilibrated to the bias placed on it before accumulating ionization readings by pre‐irradiating it with 500 mu. Due to the very small sensitive volume of the Exradin A16, ionization readings (300–400 monitor units per reading) were measured on the picocoulomb scale.

### B. Calculations

Each institution was asked to calculate the number of monitor units, using its treatment planning system, which were required to deliver 1000 cGy to the measurement point at a depth of 10 cm in water on central axis under each field size configuration. Based on the dose per monitor unit calculated and normalized to the $10\times 10{\;\text{cm}}^{2}$ value, the institution's treatment planning system‐calculated small field output factors were determined. These values were compared against the RPC measured values. The average absolute percent difference was also calculated between the RPC measured value and the institution's calculated value for each field size.

## III. RESULTS & DISCUSSION

The RPC measured and institution TPS calculated small field size dependence output factors for Varian, Elekta, and Siemens accelerators are found in Tables 1–3, respectively. In addition to the small field output factors, the variability in the values, as indicated by the standard deviations of the measured and TPS calculated values, are also shown. Tables 1–3 also list the average absolute percent differences (in square brackets) determined between the RPC and the institution's values for the Varian, Elekta and Siemens accelerators, respectively.

Varian small field output factors were measured and compared to treatment planning system calculated values for 6, 10, 15 and 18 MV X‐rays as seen in Table 1. The RPC measured output factors decreased with decreasing MLC field size, as expected. The specifics of this decrease as a function of energy were examined using an analysis of variance (ANOVA; SPSS 19;α=0.05). The most pronounced decrease with field size was for the 6 MV beam; the 6 MV output factors were significantly smaller than the output factors for any other energy for all examined field sizes (except the 18 MV beam for the $2\times 2{\;\text{cm}}^{2}$ field; p<0.05). There was no significant difference in the measured output factors between the other energies examined for any field size (except the 18 MV beam for the $2\times 2{\;\text{cm}}^{2}$ field). The spread in the RPC's measured values (standard deviation) was, on average, 1.5% (ranging from 0.8%–2.4%). The magnitude of this spread was independent of field size or energy (p>0.05). When compared to the treatment planning system‐calculated values, the RPC's measured values agreed for all field sizes and energies within 1 standard deviation. On average, the standard deviation of the planning system‐calculated values was slightly larger than the standard deviation of the measured values (2.5% for calculated vs. 1.5% for measured). The spread in the calculated values was noticeably larger for the smallest field sizes, particularly $2\times 2{\;\text{cm}}^{2}$ field size for which the average standard deviation was 3.1% and reached a maximum of 3.8%. This reflects the increased challenge in converting measured data into a commissioned beam model for very small fields, including the $2\times 2{\;\text{cm}}^{2}$ field. It also means that for individual linear accelerators, measurements more often disagreed with calculation for the smallest field sizes. This difference between the RPC's measured values and the institutions' values is also noted in Table 1 where, for the $6\times 6{\;\text{cm}}^{2}$ to $3\times 3{\;\text{cm}}^{2}$ field sizes, the average absolute percent differences (square brackets) ranged from 0.5% to 1.7%, while for the $2\times 2{\;\text{cm}}^{2}$ field size, the average absolute percent differences ranged from 1.8%–3.5% (i.e., a much larger difference). Not only was the average absolute percent difference higher for the smallest field size, but because the output factor is numerically lower (0.784–0.815), the impact on dose delivery is even greater, being nearly 5% for the 18 MV beam.

**Table 1:** The RPC‐measured and institution treatment planning system‐calculated small field size dependence output factor values for Varian machines. The values in square brackets and parentheses beneath each energy for each field size value are the average absolute percent differences and standard deviations of the values, respectively. For each energy and field size, the number of measurements (accelerators) is also shown.

	*Varian 6 MV*		*Varian 10 MV*		*Varian 15 MV*		*Varian 18 MV*	
*Field Size (cm* x cm)	*RPC*	*Institution*	*RPC*	*Institution*	*RPC*	*Institution*	*RPC*	*Institution*
10×10	1.000	1.000	1.000	1.000	1.000	1.000	1.000	1.000
6×6	**0.921**	0.929	**0.946**	0.953	**0.951**	0.950	**0.949**	0.950
	(0.013)	(0.004)	(0.017)	(0.016)	(0.008)	(0.008)	(0.011)	(0.014)
	[0.9%] (n=64)		[0.7%] (n=9)		[0.5%] (n=14)		[0.5%] (n=16)	
4×4	**0.865**	0.874	**0.900**	0.912	**0.909**	0.909	**0.902**	0.900
	(0.018)	(0.021)	(0.024)	(0.030)	(0.013)	(0.017)	(0.014)	(0.024)
	[1.3%] (n=64)		[1.3%] (n=9)		[1.1%] (n=14)		[1.1%] (n=16)	
3×3	**0.828**	0.841	**0.867**	0.875	**0.874**	0.877	**0.861**	0.856
	(0.017)	(0.025)	(0.020)	(0.025)	(0.014)	(0.019)	(0.014)	(0.027)
	[1.7%] (n=62)		[1.2%] (n=9)		[1.3%] (n=12)		[1.7%] (n=16)	
2×2	**0.786**	0.796	**0.817**	0.828	**0.803**	0.813	**0.784**	0.782
	(0.019)	(0.031)	(0.015)	(0.019)	(0.016)	(0.038)	(0.015)	(0.034)
	[2.3%] (n=55)		[1.8%] (n=11)		[2.8%] (n=10)		[3.5%] (n=15)	

Results for the Elekta accelerators (Table 2) and Siemens accelerators (Table 3) are similar to those for the Varian accelerators. The measured output factors decreased with field size. The 6 MV beams typically had a smaller output factor than the higher energy beams, although this was more pronounced for the Siemens accelerators (for which there were significant differences at all field sizes, p<0.05 based on independent samples t‐test) than for Elekta accelerators for which most of the differences were not significant (p>0.05 based on ANOVA). As with the Varian accelerators, the average measured and planning system calculated output factors agreed within 1 standard deviation for all energies and field sizes. And again, for the smallest field size there was a larger average standard deviation in the output factors for all energies when calculated by planning systems (4.5% for Siemens, 1.9% for Elekta) as compared to the measured values (0.6% for Siemens, 0.9% for Elekta). Similar to the Varian data, the average absolute percent differences (square brackets) noted for the Elekta and Siemens data (Tables 2–3) range between 0.3%–1.3% for the $6\times 6{\;\text{cm}}^{2}$ to $3\times 3{\;\text{cm}}^{2}$ field sizes; however, for the $2\times 2{\;\text{cm}}^{2}$ field size, the percent differences ranged from 1.3% to 5.8%. The percent differences and range of values for the $2\times 2{\;\text{cm}}^{2}$ field size observed for the Varian, Elekta, and Siemens data were all greater than the differences noted for the other field sizes by a factor of approximately 2–4. There was a much greater degree of variability in the percent differences for the $2\times 2{\;\text{cm}}^{2}$ field size than noted for the other field sizes.

**Table 2:** The RPC‐measured and institution treatment planning system‐calculated small field size dependence output factor values for Elekta machines. The values in square brackets and parentheses beneath each energy for each field size value are the average absolute percent differences and standard deviations of the values, respectively. For each energy and field size, the number of measurements (accelerators) is also shown.

	*Elekta 6 MV*		*Elekta 10 MV*		*Elekta 18 MV*	
*Field Size (cm x cm)*	*RPC*	*Institution*	*RPC*	*Institution*	*RPC*	*Institution*
10×10	1.000	1.000	1.000	1.000	1.000	1.000
6×6	**0.930**	0.934	**0.937**	0.940	**0.945**	0.947
	(0.010)	(0.009)	(0.004)	(0.005)	(0.002)	(0.003)
	[0.5%]		[0.7%]		[0.3%]	
	(n=18)		(n=6)		(n=5)	
4×4	**0.878**	0.888	**0.890**	0.891	**0.901**	0.918
	(0.015)	(0.027)	(0.009)	(0.010)	(0.002)	(0.039)
	[1.3%]		[0.6%]		[0.4%]	
	(n=22)		(n=8)		(n=6)	
3×3	**0.842**	0.848	**0.857**	0.862	**0.861**	0.863
	(0.012)	(0.009)	(0.003)	(0.005)	(0.003)	(0.004)
	[0.9%]		[0.6%]		[0.6%]	
	(n=17)		(n=6)		(n=4)	
2×2	**0.790**	0.796	**0.796**	0.802	**0.786**	0.798
	(0.007)	(0.010)	(0.009)	(0.008)	(0.006)	(0.019)
	[1.6%]		[1.3%]		[2.4%]	
	(n=17)		(n=6)		(n=4)	

**Table 3:** The RPC‐measured and institution treatment planning system‐calculated small field size dependence output factor values for Siemens machines. The values in square brackets and parentheses beneath each energy for each field size value are the average absolute percent differences and standard deviations of the values, respectively. For each energy and field size, the number of measurements (accelerators) is also shown.

	*Siemens 6 MV*		*Siemens 10 MV*		*Siemens 18 MV*	
*Field Size (cm X cm)*	*RFC*	*Institution*	*RPC*	*Institution*	*RFC*	*Institution*
10×10	1.000	1.000	1.000	1.000	1.000	1.000
6×6	**0.914**	0.920	**0.927**	0.935	**0.940**	0.946
	(0.008)	(0.008)	(0.003)	(0.010)	(0.005)	(0.003)
	[0.7%]		[0.9%]		[0.6%]	
	(n=13)		(n=4)		(n=4)	
4×4	**0.855**	0.863	**0.877**	0.884	**0.891**	0.896
	(0.010)	(0.009)	(0.001)	(0.012)	(0.004)	(0.003)
	[1.1%]		[1.2%]		[0.6%]	
	(n=13)		(n=4)		(n=4)	
3×3	**0.820**	0.825	**0.841**	0.850	**0.849**	0.855
	(0.008)	(0.011)	(0.001)	(0.007)	(0.003)	(0.003)
	[1.3%]		[1.1%]		[0.7%]	
	(n=13)		(n=4)		(n=4)	
2×2	**0.764**	0.757	**0.777**	0.742	**0.795**	0.779
	(0.010)	(0.042)	(0.005)	(0.079)	(0.004)	(0.015)
	[2.8%]		[5.8%^a^]		[1.9%]	
	(n=12)		(n=4)		(n=4)	

^a^ An institution value was 25% different to the RPC‐measured value. The institution corrected its data subsequent to the RPC visit.

Also of note, there were small but significant differences between the output factors from different accelerators at a given energy and field size. Agreement between accelerators would not generally be expected in this dataset for the Varian accelerator because the field definitions varied between manufacturers. While the fields defined with the Siemens and Elekta accelerators were both constructed with secondary jaws, the fields sizes defined with the Varian accelerators used the tertiary MLC with the secondary jaws defining the 10 by 10 field. Between the Siemens and Elekta accelerators, the output factors were significantly smaller for the Siemens machines at both 6 and 10 MV (p<0.05). However, disagreement between measured and calculated values was comparable regardless of machine manufacturer.

The data as shown and described in this study extend down to a $2\times 2{\;\text{cm}}^{2}$ field size. Output factors become increasingly inaccurate when measured with pinpoint ion chambers for smaller ($<2\times 2{\;\text{cm}}^{2}$) fields. The volume of air averages out the signal across the chamber, which is unlikely to be uniform in very small fields as the penumbrae comprise much of the radiation field. Furthermore, as the air cavity makes up an increasing amount of the radiation field, there is an increase in the lateral electronic disequilibrium, resulting in less dose deposition than if water existed in place of the air. Combined, these effects result in pinpoint chambers underestimating the true output for small fields by several percent (absolute difference), particularly for field sizes at or below $1\times 1{\;\text{cm}}^{2}$.^6^, ^7^, ^10^

The consistency in the output factors that was observed on accelerators of a given manufacturer may not persist to very small field sizes. Although the standard deviation of, for example, the $2\times 2{\;\text{cm}}^{2}$ measured data for the Varian 6 MV beam was very small, it may be expected to increase with decreasing field size. For fields smaller than $2\times 2{\;\text{cm}}^{2}$, the size of the direct source affects dosimetry parameters such as output factor, beam profiles, and percent depth doses.^3^, ^4^ This is a problem because it has also been observed that different accelerators, or different energies on the same accelerator, have different source sizes.^(11)^ Consequently, the development of a standard dataset for output factors of field sizes smaller than $2\times 2{\;\text{cm}}^{2}$ may be impossible.

The data presented here are intended to serve as a guide to help potentially identify gross errors in the commissioning of small field output factor data. Because different machines may have different small field properties, particularly for very small fields, field size dependent output factors must be established through accurate individual accelerator‐specific measurements and treatment planning computer beam model parameterization. An example of this is for the Varian/Pinnacle planning system combination. To properly model the beams for small MLC defined field sizes, one must modify the planning system software's Gaussian parameters that model the head scatter. In order to achieve accurate dose calculations, uniformity of the scatter effect correction factor (OFc) among the various field sizes has to be maintained by adjusting the Gaussian flattening filter scatter source. Once the model has been adjusted, the beam model's dose calculations for all conditions must be reevaluated against measurements.

Regardless of the ability to model a photon beam's dosimetric characteristics in the treatment planning system from an institution's measurements, if the measurements were taken inappropriately, then the best modeling will result in erroneous values. The need for an institution to make accurate small field size measurements is crucial for accurate treatment planning system calculations. Institutions must use extreme caution when selecting the dosimeter to ensure that its measurement volume is adequate for the small field sizes in order to reduce the partial volume effect. In addition to the dosimeter size, placement of the dosimeter within the field must be such that the center of the dosimeter is placed at the location of the peak signal, since the dose profiles for these very small fields is typically not flat but tend to be peaked and rounded. The AAPM's Task Group 155 is currently working on a report to assist the medical physicist in making appropriate and accurate small field size output factor measurements.

However, having said this, this work also found that there was a relatively high degree of consistency between accelerators in terms of the field size dependent output factors as measured by the RPC. Therefore, for this range of field sizes, the compiled data here provide an independent set of measured data that might alert an institution to any gross errors in commissioning. It is also important to consider that IMRT delivery includes many steps beyond basic dosimetry. Proper commissioning and quality assurance should include an assessment of the IMRT delivery process from end to end, as well as the individual dosimetry parameters required to determine an IMRT dose distribution.

## IV. CONCLUSIONS

The data presented here provide a consistent dataset for small field output factors that can be used as a redundant QA check of a treatment planning system dosimetry data for small‐field treatments. The RPC's measured values have a small uncertainty (standard deviation <2%), while the values calculated from the various planning systems and their beam models had a greater uncertainty, especially for the smallest field sizes. As more institutions model their treatment beams in their planning systems to deliver IMRT or stereotactic treatments that use small field size segments, they are required to make small field size measurements that pose unique challenges. A QA dataset against which the institution can compare its measured or calculated values is needed to ensure accurate IMRT dose delivery by identifying discrepancies prior to any patients being treated.

## ACKNOWLEDGMENTS

This work was supported by Public Health Service Grant CA10953 awarded by the National Cancer Institute, United States Department of Health and Human Services. We would like to thank Elizabeth Siller for typing the many drafts of this manuscript.
