# Erratum: “The Radiological Physics Center's standard dataset for small field size output factors”

**DOI:** 10.1120/jacmp.v15i2.4757

**Published:** 2014-03-06

After the publication of the manuscript titled “The Radiological Physics Center's standard dataset for small field size output factors,” an error was discovered in the data used to compile the values listed in Table 1.

The error consisted of the inadvertent addition of RPC‐measured and institution‐calculated values for Varian machines that did not correspond to the measurement geometry described in the Materials and Methods section of the manuscript. The incorrect data have been removed from the analysis. Additionally, subsequent additional correct data have been added to generate a new Table 1 (shown below). The specific values for the small field size‐dependent output factors have changed slightly for the Varian accelerator, but all of the original findings and conclusions remain valid.

The new corrected Table 1 values have resulted in the following changes (italics) to the text of the manuscript.

## ORiGiNAL CiTATiON

Followill DS, Kry SF, Qin L, Lowenstein J, Molineu A, Alvarez P, Aguirre JF, Ibbott GS. The Radiological Physics Center's standard dataset for small field size output factors. J Appl Clin Med Phys. 2012;13(5):3962.

## ABSTRACT

The RPC‐measured output factors are tabulated and are reproducible with standard deviations (SD) ranging from *0.1% to 1.5%*, while the institutions' calculated values had a much larger SD range, ranging up to *7.9%.*

## MATERiALS AND METHODS

### A. Measurements

Measurements were made on modern Varian (n=136) (Varian Medical Systems, Palo Alto, CA), Elekta (n=22) (Elekta, Stockholm, Sweden), and Siemens (n=10) (Siemens AG, Munich, Germany) accelerators that included an MLC and had indicated clinical use of IMRT.

## RESULTS AND DiSCUSSiON

The spread in the RPC's measured values (standard deviation) was, on average, *0.6% (ranging from 0.2%—1.1%).* The magnitude of this spread was independent of field size or energy (p>0.05). When compared to the treatment planning system‐calculated values, the RPC's measured values agreed for all field sizes and energies within *2 standard deviations for the 15 and 18 MV beams, with the exception of the 18 MV* $4\times 4\;{\text{cm}}^{2}$ *value. However, the comparison between the RPC‐measured and TPS‐calculated values for the 6 and 10 MV beams tended to exceed the RPC measurements by more than 2 standard deviations for the majority of the field sizes measured.* On average, the standard deviation of the planning system‐calculated values was slightly larger than the standard deviation of the measured values *(1.3% for calculated vs. 0.6% for measured).* The spread in the calculated values was noticeably larger for the smallest field sizes, particularly $2\times 2\;{\text{cm}}^{2}$ field size, for which the average standard deviation was *2.4% and reached a maximum of 4.0%.* This reflects the increased challenge in converting measured data into a commissioned beam model for very small fields, including the $2\times 2\;{\text{cm}}^{2}$ field. It also means that for individual linear accelerators, measurements more often disagreed with calculation for the smallest field sizes. This difference between the RPC's measured values and the institutions' values is also noted in Table 1 where, for the $6\times 6\;{\text{cm}}^{2}$ field size, the average absolute percent differences (square brackets) *ranged from 0.4% to 0.8%*, while for the $2\times 2\;{\text{cm}}^{2}$ field size, the average absolute percent differences *ranged from 1.6%‐2.9%* (i.e., a larger difference). Not only was the average absolute percent difference higher for the smallest field size but, because the output factor is numerically lower (0.804−0.824), the impact on dose delivery is even greater, *being nearly 4% for the 6 MV beam.*

**Table 1:** The RPC‐measured and institution treatment planning system‐calculated small field size dependence output factor values for Varian machines. The values in square brackets and parentheses beneath each energy for each field size value are the average absolute percent differences and standard deviations of the values, respectively. For each energy and field size, the number of measurements (accelerators) is also shown

*Field Size* (cm×cm)	*Varian 6MV RPC Institution*		*Varian 10 MV RPC Institution*		*Varian 15 MV RPC Institution*		*Varian 18 MV RPC Institution*	
10×10	1.000	1.000	1.000	1.000	1.000	1.000	1.000	1.000
6×6	**0.938**	0.945	**0.959**	0.964	**0.963**	0.964	**0.969**	0.971
	(0.005)	(0.008)	(0.002)	(0.003)	(0.005)	(0.007)	(0.003)	(0.006)
	[0.8%]		[0.5%]		[0.4%]		[0.5%]	
	(n=127)		(n=22)		(n=25)		(n=41)	
4×4	**0.886**	0.900	**0.916**	0.927	**0.927**	0.928	**0.928**	0.933
	(0.006)	(0.013)	(0.006)	(0.010)	(0.006)	(0.009)	(0.002)	(0.009)
	[1.8%]		[1.2%]		[0.7%]		[0.9%]	
	(n=122)		(n=25)		(n=32)		(n=37)	
3×3	**0.851**	0.870	**0.880**	0.894	**0.892**	0.894	**0.884**	0.891
	(0.007)	(0.012)	(0.006)	(0.008)	(0.006)	(0.012)	(0.005)	(0.019)
	[2.4%]		[1.6%]		[0.9%]		[1.4%]	
	(n=123)		(n=25)		(n=31)		(n=41)	
2×2	**0.804**	0.825	**0.823**	0.838	**0.824**	0.823	**0.806**	0.804
	(0.008)	(0.020)	(0.005)	(0.015)	(0.011)	(0.040)	(0.007)	(0.020)
	[2.9%]		[2.0%]		[2.2%]		[1.6%]	
	(n=136)		(n=23)		(n=33)		(n=40)	

